# Correction: Fluorescent reporter plasmids for single-cell and bulk-level composition assays in *E. faecalis*

**DOI:** 10.1371/journal.pone.0329532

**Published:** 2025-08-01

**Authors:** Kelsey M. Hallinen, Keanu A. Guardiola-Flores, Kevin B. Wood

The author Kelsey M. Hallinen should be noted as the corresponding author for this work. Kelsey M. Hallinen’s email address is: hallinen@uoregon.edu
