# Supplementary material for: Fluorescent reporter plasmids for single-cell and bulk-level composition assays in E. faecalis
Source: PLoS One. 2020 May 5;15(5):e0232539. doi: 10.1371/journal.pone.0232539 (PMC7199960; doi:10.1371/journal.pone.0232539)
Supplement: S1 Table — (PDF) [file pone.0232539.s001.pdf]

**Name**	**Sequence**	**Target**
pBSU 101-For	5'-AGC GGC CGC GAC TCT AGA G-3'	pBSU 101 backbone forward
pBSU 101-Rev	5'-GGT GGC GAC CGG TAC CCG-3'	pBSU 101 backbone reverse
BFP-pBSU 101 For	5'-CCC GGG TAC CGG TCG CCA CCA TGG TGT CTA AGG GCG AAG-3'	BFP and pBSU 101 overlap forward
BFP-pBSU 101 Rev	5'-ACT CTA GAG TCG CGG CCG CTA TTA AGC TTG TGC CCC AG-3'	BFP and pBSU 101 overlap reverse
CL CFP-pBSU 101 For	5'-CCC GGG TAC CGG TCG CCA CCA TGT CGT CTG GTG CCA AAT TG-3'	CFP and pBSU 101 overlap forward
CL CFP-pBSU 101 Rev	5'-ACT CTA GAG TCG CGG CCG CTT TAC TGA TAC GTG TCC AGA TCA AC-3'	CFP and pBSU 101 overlap reverse
Cratchit YFP®-pBSU 101 For	5'-CCC GGG TAC CGG TCG CCA CCA TGA CGG CAT TGA CGG AAG-3'	YFP and pBSU 101 overlap forward
Cratchit YFP®-pBSU 101 Rev	5'-ACT CTA GAG TCG CGG CCG CTT TAG CGA TAC GTC TCC AGG-3'	YFP and pBSU 101 overlap reverse
Yeti YFP®-pBSU 101 For	5'-CCC GGG TAC CGG TCG CCA CCA TGA CGG CAT TGA CGG AAG-3'	YFP and pBSU 101 overlap forward
Yeti YFP®-pBSU 101 Rev	5'-ACT CTA GAG TCG CGG CCG CTT TAG CGA TAC GTC TCC AGG-3'	YFP and pBSU 101 overlap reverse
