# Supplementary material for: Fluorescent reporter plasmids for single-cell and bulk-level composition assays in E. faecalis
Source: PLoS One. 2020 May 5;15(5):e0232539. doi: 10.1371/journal.pone.0232539 (PMC7199960; doi:10.1371/journal.pone.0232539)
Supplement: S2 Table — (PDF) [file pone.0232539.s002.pdf]

Name	Sequence	Target
Comet GFP®-pBSU 101 For	5'-CCC GGG TAC CGG TCG CCA CCA TGA CGG CAT TGA CGG AAG-3'	GFP and pBSU 101 overlap forward
Comet GFP®-pBSU 101 Rev	5'-ACT CTA GAG TCG CGG CCG CTT TAA CGG TAA GTT TCC AGG TC-3'	GFP and pBSU 101 overlap reverse
Dasher GFP®-pBSU 101 For	5'-CCC GGG TAC CGG TCG CCA CCA TGA CGG CAT TGA CGG AAG-3'	GFP and pBSU101 overlap forward
Dasher GFP®-pBSU 101 Rev	5'-ACT CTA GAG TCG CGG CCG CTT TAC TGA TAC GTG TCC AGA TC-3'	GFP and pBSU 101 overlap reverse
Rud RFP-pBSU 101 For	5'-CCC GGG TAC CGG TCG CCA CCA TGT CCC TGT CGA AAC AAG-3'	RFP and pBSU 101 overlap forward
Rud RFP-pBSU 101 Rev	5'-ACT CTA GAG TCG CGG CCG CTT TAC GTT TCT TTA ACG TCG AC-3'	RFP and pBSU 101 overlap reverse
Fresno RFP®-pBSU 101 Rev	5'-CCC GGG TAC CGG TCG CCA CCA TGA ATA GCC TGA TTA AAG AGA ATA TG-3'	RFP and pBSU 101 overlap forward
Fresno RFP®-pBSU 101 Rev	5'-ACT CTA GAG TCG CGG CCG CTT TTG TAC AGT TCG TCC ATA C-3'	RFP and pBSU 101 overlap reverse
