# Supplementary material for: Fluorescent reporter plasmids for single-cell and bulk-level composition assays in E. faecalis
Source: PLoS One. 2020 May 5;15(5):e0232539. doi: 10.1371/journal.pone.0232539 (PMC7199960; doi:10.1371/journal.pone.0232539)
Supplement: S3 Table — (PDF) [file pone.0232539.s003.pdf]

Name	Sequence	Target
Opt BFP-pBSU 101 For	5'-CCC GGG TAC CGG TCG CCA CCA TGG TTT CAA AAG GTG AAG-3'	Opt BFP and pBSU 101 overlap forward
Opt BFP-pBSU 101 Rev	5'-ACT CTA GAG TCG CGG CCG CTG TTT AAT TTG TGA CCT AAT TTT G-3'	Opt BFP and pBSU 101 overlap reverse
Opt Rudolph RFP®-pBSU 101 For	5'-CCC GGG TAC CGG TCG CCA CCA TGT CAT TAT CAA AAC AAG TTT TAC-3'	RFP and pBSU 101 overlap forward
Opt Rudolph RFP®-pBSU 101 Rev	5'-ACT CTA GAG TCG CGG CCG CTT TAT GTT TCT TTA ACA TCA ACT G-3'	RFP and pBSU 101 overlap reverse
pBSU 101-Section1 For	5'-CGG AGC CTA TGG AAA AAC GCC AGC AAC GCG GCC TTT TTA CG-3'	Overlap: section 1 and section 2 forward
pBSU 101-Section1 Rev	5'-ACT CTA GAG TCG CGG CCG CTG GTG GCG ACC GGT GGT ACC CG-3'	Overlap: section 1 and section 2 reverse
pBSU 101-Section2 For	5'-AGC GGC CGC GAC TCT AGA-3'	pBSU 101 section 2 forward
pBSU 101-Section2 Rev	5'-GCG TTT TTC CAT AGG CTC-3'	pBSU 101 section 2 reverse

For the no color plasmid, PCR was used to create the backbone in two separate pieces and Gibson Assembled back together, without the color sequence.
