# Supplementary material for: Fluorescent reporter plasmids for single-cell and bulk-level composition assays in E. faecalis
Source: PLoS One. 2020 May 5;15(5):e0232539. doi: 10.1371/journal.pone.0232539 (PMC7199960; doi:10.1371/journal.pone.0232539)
Supplement: S4 Table — (PDF) [file pone.0232539.s004.pdf]

Color	Excitation (nm)	Emission (nm)
mTagBFP2	401	440-530
CindyLou CFP®	400	450-580
Yeti YFP®	500	520-580
Cratchit YFP®	500	520-580
Comet GFP®	480	500-570
Dasher GFP®	480	500-570
EGFP	470	490-560
Rudolph RFP®	545	565-625
Fresno RFP®	545	565-625

Emission spectra were taken in the range listed with a step size of 5 nm.
