# Supplementary material for: Fluorescent reporter plasmids for single-cell and bulk-level composition assays in E. faecalis
Source: PLoS One. 2020 May 5;15(5):e0232539. doi: 10.1371/journal.pone.0232539 (PMC7199960; doi:10.1371/journal.pone.0232539)
Supplement: S5 Table — (PDF) [file pone.0232539.s005.pdf]

Color	Excitation (nm)	Emission (nm)
mTagBFP2	401	440-465
CindyLou CFP®	400	495-520
Cratchit YFP®	500	525-550
Dasher GFP®	480	510-535
Rudolph RFP®	545	585-610

Emissions spectra were taken at a total of 6 equally spaced wavelengths spanning the range above.
