# Supplementary material for: Fluorescent reporter plasmids for single-cell and bulk-level composition assays in E. faecalis
Source: PLoS One. 2020 May 5;15(5):e0232539. doi: 10.1371/journal.pone.0232539 (PMC7199960; doi:10.1371/journal.pone.0232539)
Supplement: S6 Table — (PDF) [file pone.0232539.s006.pdf]

Color	Excitation Laser	Laser Power (mW)	Laser Attenuation (%)	Protocol
mTagBFP2	405 nm	5	5.0	mBFP
CindyLou CFP®	405 nm	5	5.0	mBFP
Yeti YFP®	488 nm	10	1.0	Lucifer Yellow
Cratchit YFP®	488nm	10	1.0	Lucifer Yellow
Comet GFP®	405 nm	5	5.0	EGFP
Dasher GFP®	488 nm	10	0.5	EGFP
EGFP	488 nm	10	0.5	EGFP
Rudolph RFP®	555 nm	10	5.0	mStrawberry
Fresno RFP®	555 nm	10	5.0	mStrawberry

Values are based on pre-loaded protocols in the Zeiss LSM700 software for similar fluorescent proteins.
